# Wideband TDoA Positioning Exploiting RSS-Based Clustering

**DOI:** 10.3390/s23125772

**Published:** 2023-06-20

**Authors:** Andreas Fuchs, Lukas Wielandner, Daniel Neunteufel, Holger Arthaber, Klaus Witrisal

**Affiliations:** 1 Signal Processing and Speech Communication Laboratory, Graz University of Technology, 8010 Graz, Austria; 2 Christian Doppler Laboratory for Location-Aware Electronic Systems, 8010 Graz, Austria; 3 Institute of Electrodynamics, Microwave and Circuit Engineering, TU Wien, 1040 Vienna, Austria

**Keywords:** indoor positioning, Internet of Things, CRLB, AoA, ToA, sensor fusion, RSS, wideband

## Abstract

The accuracy of radio-based positioning is heavily influenced by a dense multipath (DM) channel, leading to poor position accuracy. The DM affects both time of flight (ToF) measurements extracted from wideband (WB) signals—specifically, if the bandwidth is below 100 MHz—as well as received signal strength (RSS) measurements, due to the interference of multipath signal components onto the information-bearing line-of-sight (LoS) component. This work proposes an approach for combining these two different measurement technologies, leading to a robust position estimation in the presence of DM. We assume that a large ensemble of densely-spaced devices is to be positioned. We use RSS measurements to determine “clusters” of devices in the vicinity of each other. Joint processing of the WB measurements from all devices in a cluster efficiently suppresses the influence of the DM. We formulate an algorithmic approach for the information fusion of the two technologies and derive the corresponding Cramér-Rao lower bound (CRLB) to gain insight into the performance trade-offs at hand. We evaluate our results by simulations and validate the approach with real-world measurement data. The results show that the clustering approach can halve the root-mean-square error (RMSE) from about 2 m to below 1 m, using WB signal transmissions in the 2.4 GHz ISM band at a bandwidth of about 80 MHz.

## 1. Introduction

### 1.1. State of the Art

Radio-based indoor localization is an increasingly important research topic, as many modern electronic devices are dependent on robust and accurate position information to provide location-dependent services and applications. Exemplary applications include positioning in retail scenarios guiding costumers to products they are looking for, tracking medical devices in healthcare environments, providing guests of museums with accurate position-dependent interactive tours, tracking articles in warehouses and logistic centers, and many more [1,2,3,4,5,6,7,8].

Current state-of-the-art algorithms focus mostly on one measurement method, which can, for example, include received signal strength (RSS) measurements from multiple devices to each other, wideband (WB) measurements to infrastructures such as wireless modems and other equipment or measurements with higher bandwidths, e.g., ultra-wideband (UWB).

These multiple measurement methods have their own advantages and disadvantages, as the technology imposes direct limitations. Measurements of RSS values are, for example, relatively easy to acquire, but the information content of a single measurement is low, and thus, a single measurement provides only marginal positional information. Therefore, a need arises for a large number of (independent) measurements to increase the positional information to an acceptable level, and additionally, a significant number of fixed “anchor nodes” are necessary for reference [9]. Other technologies, for example, time difference of arrival (TDoA)-based localizations utilizing WB measurements in the industrial, scientific, and medical (ISM) bands (i.e., 80 MHz at 2.4 GHz), provide much more information with a single measurement, but additional WB anchor infrastructure is necessary—so-called access points (APs), similar to [10] (Chapter 6). In this case, the devices and APs need more complicated radio chips to send and receive higher bandwidth signals. Chips providing even higher bandwidths are increasingly cost-intensive and power-hungry, and thus, not economical for many applications.

All of the previously mentioned radio-based localization technologies have in common that multipath-propagation influences the measurements [11], which can negatively affect the results. For bandwidths smaller than 100 MHz, in which typical indoor scenario multipath components can not be discerned anymore, the interfering dense multipath component (DMC) leads to diminished performance [12].

Algorithms developed in recent years focus on many different approaches, including machine learning [13] or classical signal processing [14,15], but focus mostly on single measurement methods. For many of these approaches, essential performance bounds such as the Cramér-Rao lower bound (CRLB) are also derived [16,17,18,19,20].

Combining multiple measurement methods allows us to use complimentary gains from each method, but increases the complexity of the system architecture and of the algorithms [21]. Thus, it must be regarded when developing new approaches. Some research was conducted to fuse multiple localization and measurement methods [1,22]. These algorithms combine position estimates, but do not fuse measurement data directly. Other existing methods incorporate maximum likelihood estimates for time of arrival (ToA), angle of arrival (AoA), and RSS, but those methods rely on fusing multiple measurements from a single node [23,24], they do not fuse the information of multiple nodes. Furthermore, there is no research yet that focuses on the derivation of a CRLB for such fused algorithms. Other methods for data fusion of multiple measurements incorporate machine learning, for example, methods based on channel state information [25,26] or support vector regression [27,28]. Machine learning algorithms need training data to work, which is not necessary for our proposed algorithm.

### 1.2. Concept

This work proposes a method that combines WB time of flight (ToF) and AoA measurements of multiple nodes in an indoor scenario. To overcome the limitations of single WB measurements, RSS measurements are collected between the nodes, to determine those in the vicinity of each other. By selecting the WB measurements of those nodes, we obtain access to multiple realizations of the interfering dense multipath component (DMC) and thus an additional information gain for every measurement.

This is achieved by an approach we call clustering, where RSS measurements are used to find the nearest nodes, for which WB measurements are then processed jointly.

**Figure 1:**
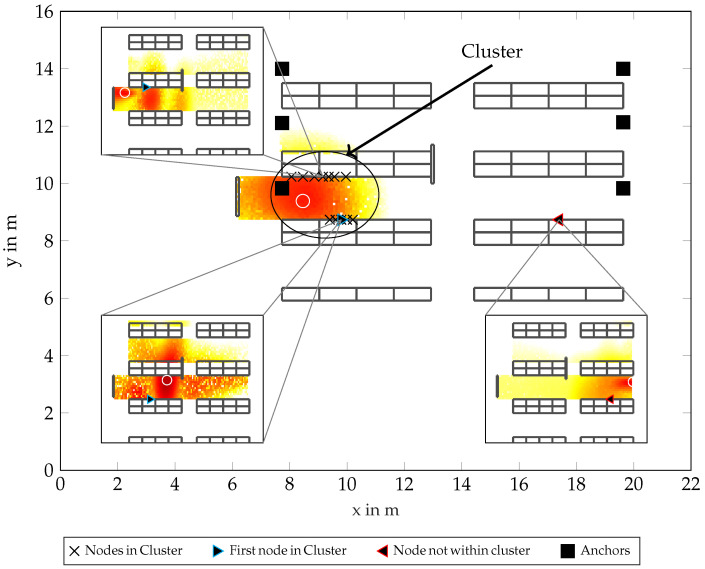
Visualization of the RSS-based clustering. Excerpts show single likelihoods over a floorplan; overall graphic shows a combined likelihood from a set of 15 nodes.

In the following, we describe likelihoods for node and cluster positions, which describe regions with likelihoods of a node being at one position. In Figure 1, this concept is visualized in a typical warehouse scenario, incorporating multiple shelves defining aisles, nodes placed on these shelves, and multiple anchors over the room.

Here, the likelihood of a room with measurements on six antenna-arrays, each incorporating two antennas, can be seen, where red regions show a large likelihood for a node to be in a position.

The antenna arrays allow for coherent processing, yielding information on the AoA. Different anchor positions allow for a TDoA positioning approach. In the upper-left subplot, a single likelihood for WB measurements is shown. One can see that the resulting likelihood is multi-modal, and the position with the largest likelihood (shown as a white circle) is at one of the false modes. This multi-modality stems from the estimation of false peaks in the time-domain, which are caused by multipath components dominating over the line-of-sight (LoS). The real position is shown as a blue triangle. The same can be seen in the lower-left subplot, for a node in the vicinity, showing similar effects. In addition, this measurement suffered from a poor signal-to-noise ratio (SNR), which broadens the likelihood in all spatial directions. The lower-right subplot shows a single node far away from the other two examples, for which the estimation works rather poorly. These cases can be mitigated by the clustering approach.

To improve the positioning accuracy and reliability of a single node, multiple nodes can be clustered, and thus a joint likelihood can be computed, which mitigates the uncertainties. However, the strategy for clustering is not clear, as there is no previous information from WB measurements alone. Here, RSS measurements can be used, giving us a list of nodes that are likely near a node in a cluster (which is seen as a defining “first” node for the cluster), seen again as a blue triangle. All other nodes within this cluster are shown as black crosses. Now, the joint likelihood seen in Figure 1 for the whole room is for a combination of all WB measurements within this cluster, applying our proposed algorithm. This shows a single defined mode, where the maximum is the estimated position for the defining node. Note that these nodes can be positioned at different heights, which the algorithm takes into account by a three-dimensional formulation. This approach would also be applicable to multiple measurements of a single node at different positions near each other, but this work focuses on static scenarios.

In order to assess the achievable performance, the CRLB for the cluster center incorporating the DMC was evaluated. The algorithm treats measurements from multiple nodes as different realizations of a single node; thus, a correction factor is introduced. This factor regards the information loss from cluster nodes being in different positions. Therefore, we have to introduce a bias term on top of the CRLB since the algorithm leads to the estimation of the cluster center, which differs from the true position of the node. This is verified by simulations of increasing complexity and by a real-world measurement campaign.

### 1.3. Contribution

The contributions of this work are the following:

- A concept for the information fusion of WB TDoA and AoA measurements exploiting RSS-based clustering of multiple agent nodes to jointly process their position information.
- A maximum likelihood estimation-based algorithm for the mentioned concept.
- An efficient implementation of the proposed algorithm using a particle-based estimator.
- A derivation of estimation performance bounds for this concept incorporating:
  – Results for a correction factor describing the loss of information from large clusters.
  – Derivation of the CRLB incorporating this correction factor and information gain from multiple measurements.
  – Introduction of a biased lower bound attributing to the performance losses when estimating a single node within a cluster.
- Numeric evaluations of these bounds, showing the influence of parameters such as the number of nodes in the cluster, size of the cluster, and positions of nodes within a cluster. Specifically, we analyze the performance bounds for:
  – Single node positions, validating the information gain.
  – Increasing node distances for two nodes, validating the biased lower bound.
  – A fully synthetic measurement scenario with nodes over a simulated room with shelves, validating the data fusion concept.
  – The same scenario, incorporating real RSS measurements for clustering of adjacent nodes, but keeping synthetic WB measurements for positioning, validating the impact of realistic clustering with RSS data.
- A verification of the theoretical results with real-world measurement data for both WB and RSS, showing that the algorithm is applicable to real scenarios.

### 1.4. Paper Outline

The paper is structured as follows: Section 2 defines the overall notation. The signal model for the WB-measurements and the resulting likelihood for single measurements to one agent node are presented in Section 3. In Section 4, we describe the clustering approach in detail and the conditions that have to be met to allow this approach. In Section 5 we derive the CRLB for our system model, additionally focusing on the information loss dependent on the cluster geometry. Section 6 focuses on evaluations of both synthetic scenarios and real-world measurements, verifying performance. Lastly, a conclusion can be found in Section 7. Additional insights and detailed derivations are shown in Appendix A, Appendix B and Appendix C.

## 2. Notation

Column vectors are denoted by boldface lower-case letters, and matrices are denoted by boldface upper-case letters. The probability density function (PDF) of a random variable is denoted as f(x). For any vector x, we denote the transpose as $x^{T}$, the Hermitian transpose as $x^{H}$, the Euclidian norm as ∥x∥, the mean over all elements in the vector as $\bar{x}$, the expectation operator as $E_{i}\left[x\right]$ in dimension *i* (where dimension *i* denotes the dimension along the expectation operator is calculated), and the complex conjugate as $x^{*}$. The calligraphic notation L denotes a set, other usages of calligraphic fonts are described at their first occurrence. ${\bar{\left\{x_{l}\right\}}}_{L}$ denotes the mean operator over all vectors $x_{l}$ for *l* in the set L. Furthermore, we introduce a trace operator as tr{X} for a matrix X and det(X) as it’s determinant. Sub-matrices of a matrix X are written with their corresponding superscripts without parentheses, i.e., $X^{i,i}$. A superscript with brackets $X^{\left(i\right)}$ is used to denote a matrix designated by index *i* (notably used for matrices corresponding to a single anchor *m*) and ${\left[X\right]}_{3\times 3}$ is the upper-left 3×3 sub-block of a matrix. Real and imaginary parts of *x* are denoted as Rx and Ix, respectively.

## 3. Signal Model

The system setup consists of *L* transmitting nodes that are located at positions $p_{l}={\left[x_{l},y_{l},z_{l}\right]}^{T}\in R^{3},\forall l\in \left\{1,\ldots ,L\right\}$ and receiving antennas at positions $p_{m,k}\in R^{3},\forall m\in \left\{1,\ldots ,M\right\},\forall k\in \left\{1,\ldots ,K\right\}$, where index *k* describes the antennas within each anchor, and index *m* describes the anchor. The number of anchors and antennas per anchor are described by *M* and *K*, respectively. The radio channel from the *l*-th transmitting node to the *k*-th receiving antenna of anchor *m* is given as

(1) $$h_{l,m,k}\left(\tau ;p_{l}\right)=\alpha_{l,m}\delta \left(\tau -\tau_{m,k}\left(p_{l}\right)\right)+\nu_{l,m,k}\left(\tau \right)$$

with propagation delay $\tau_{m,k}\left(p_{l}\right)=\frac{1}{c}∥p_{l}-p_{m,k}∥$ and the complex amplitude $\alpha_{l,m}$ of the received LoS signal from node *l* to anchor *m*. The DMC is described by a zero-mean complex Gaussian random process. With the assumption of uncorrelated scattering, the auto-correlation of the random process $\nu_{l,m,k}\left(\tau \right)$ is defined as

(2) $$E\left[\nu_{l,m,k}\left(\tau \right)\nu_{l^{\prime },m^{\prime },k^{\prime }}^{*}\left(\tau^{\prime }\right)\right]=S_{\nu }\left(\tau -\tau_{m}\left(p_{l}\right),{\tilde{\nu }}_{l,m}\right)\delta \left(\tau -\tau^{\prime }\right)\delta \left[l-l^{\prime }\right]\delta \left[m-m^{\prime }\right]\delta \left[k-k^{\prime }\right],$$

where $\tau_{m}\left(p_{l}\right)=\frac{1}{c}\left(∥p_{l}-p_{m}∥\right)$ is a mean delay per array *m*, with $p_{m}=\bar{\left\{p_{m,1},\cdots ,p_{m,K}\right\}}$ being the mean antenna position. The delay power spectrum (DPS) $S_{\nu }\left(\tau -\tau_{m}\left(p_{l}\right),{\tilde{\nu }}_{l,m}\right)$ is defined later in this section. With the assumption that every node *l* is transmitting a baseband signal s(t) at frequency $f_{c}$, the received signal at anchor *m* is described by

(3) $$r_{l,m,k}\left(t\right)={\tilde{\alpha }}_{l,m,k}s\left(t-\tau_{m,k}\left(p_{l}\right)-\epsilon_{l}\right)+\int s\left(t-\tau \right)\nu_{l,m,k}\left(\tau +\epsilon_{l}\right)d\tau +w_{l,m,k}\left(t\right),$$

with a complex amplitude ${\tilde{\alpha }}_{l,m,k}=\alpha_{l,m}e^{-j2\pi f_{c}\left(\tau_{m,k}\left(p_{l}\right)+\epsilon_{l}\right)}$ that accounts for the phase shift at antenna *k*, $\epsilon_{l}$ is the transmit time of node *l*, and $w_{l,m,k}\left(t\right)$ is the noise modeled as AWGN with double-sided power spectral density (PSD) $N_{0}/2$. With this, we can describe the sampled and stacked received signals as

(4) $$r_{l,m}=s_{l,m}\left(p_{l},\epsilon_{l}\right)\alpha_{l,m}+w^{l,m}\in C^{N_{s}K\times 1},$$

where $r_{l,m}={\left[r_{l,m,1}^{T},\cdots ,r_{l,m,K}^{T}\right]}^{T}$. Additive noise resulting from the DMC and additive white Gaussian noise (AWGN) is described within the noise vector $w^{l,m}$. The baseband-signal vector is described as

(5) $$\begin{array}{cc} s_{l,m}\left(p_{l},\epsilon_{l}\right)= & [e^{-j2\pi f_{c}\left(\tau_{m,1}\left(p_{l}\right)+\epsilon_{l}\right)}s{\left(\tau_{m}\left(p_{l}\right)+\epsilon_{l}\right)}^{T},\cdots ,\\ & \;\;e^{-j2\pi f_{c}\left(\tau_{m,K}\left(p_{l}\right)+\epsilon_{l}\right)}s{\left(\tau_{m}\left(p_{l}\right)+\epsilon_{l}\right)}^{T}{]}^{T} \end{array}$$

where $s\left(\tau \right)\in C^{N_{s}\times 1}={\left[s\left(-\tau \right),s\left(\tau +T_{s}\right),\cdots ,s\left(-\tau +\left(N_{s}-1\right)T_{s}\right)\right]}^{T}$ is a sampled version of s(t−τ). Note that $T_{s}$ is the sampling time interval. This is a conventional “wideband” phased-array signal model with identical envelopes and phase shifts for the AoA.

The covariance matrix ${\left[C^{l,m}\right]}_{k}$ describes the noise vector $w^{l,m}$ and is the sampled noise covariance of the AWGN and DMC. We introduce a covariance matrix for every array element *k* as ${\left[C^{l,m}\right]}_{k}={\left[C_{\nu }^{l,m}\right]}_{k}+{\left[C_{w}^{l,m}\right]}_{k}\in C^{N_{s}\times N_{s}}$, where ${\left[C_{w}^{l,m}\right]}_{k}=\sigma_{l,m}^{2}I$, with I being the identity matrix of according dimensions and noise variance $\sigma_{l,m}^{2}=N_{0}/T_{s}$. The covariance for the DMC is described as

(6) $${\left[C_{\nu }^{l,m}\right]}_{k}=\int S_{\nu }\left(\tau -\tau_{m}\left(p_{l}\right)-\epsilon_{l};{\tilde{\eta }}_{l,m}\right)s\left(\tau \right)s{\left(\tau \right)}^{H}d\tau ,$$

where ${\tilde{\eta }}_{l,m}$ are parameters describing the shape of the DPS. Now, assuming that the DMC is a Gaussian process [16,29], the likelihood function of the model for a single node and antenna array equates to

(7) $$f_{l,m}\left(r_{l,m}|p_{l},\epsilon_{l},\eta_{l,m},\alpha_{l,m}\right)=\frac{e^{-{\left(r_{l,m}-s_{l,m}\left(p_{l},\epsilon_{l}\right)\alpha_{l,m}\right)}^{H}{\left(C^{l,m}\right)}^{-1}\left(r_{l,m}-s_{l,m}\left(p_{l},\epsilon_{l}\right)\alpha_{l,m}\right)}}{\pi^{N_{s}K}\det\left(C^{l,m}\right)},$$

with parameter vector $\eta_{l,m}={\left[\sigma_{l,m}^{2},\;{{\tilde{\eta }}_{l,m}}^{T}\right]}^{T}$ and $C^{l,m}$ being a block diagonal matrix described by the *k*-th matrices ${\left[C^{l,m}\right]}_{k}$ for every array element. To obtain a joint likelihood for a single node, the factorization of these likelihoods equates to

(8) $$f_{l}\left(r_{l}|p_{l},\epsilon_{l},\eta_{l},\alpha_{l}\right)=\prod_{m=1}^{M}f_{l,m}\left(r_{l,m}|p_{l},\epsilon_{l},\eta_{l,m},\alpha_{l,m}\right),$$

which assumes independence of the DMC and AWGN between anchors. Here, $r_{l}={\left[r_{l,1}^{T},\cdots ,r_{l,M}^{T}\right]}^{T}$ is a stacked receive vector, $\alpha_{l}={\left[\alpha_{l,1},\cdots ,\alpha_{l,M}\right]}^{T}$ are the stacked LoS amplitudes and $\eta_{l}={\left[{\eta_{l,1}}^{T},\cdots ,{\eta_{l,M}}^{T}\right]}^{T}$ is a stacked parameter vector. Lastly, we introduce the DPS $S_{\nu }\left(\tau ;\tilde{\eta }\right)$ similar to [12,29] as

(9) $$S_{\nu }\left(\tau ;\tilde{\eta }\right)=\Omega_{1}\frac{\gamma_{f}+\gamma_{r}}{{\gamma_{f}}^{2}}e^{-\tau /\gamma_{f}}\left(1-e^{-\tau /\gamma_{r}}\right)\Sigma \left(\tau \right),$$

with $\tilde{\eta }={\left[\Omega_{1},\gamma_{f},\gamma_{r}\right]}^{T}$, which corresponds to a normalized power of the DMC of $\Omega_{1}$, a fall time for the process $\gamma_{f}$, and a rise time $\gamma_{r}$. Furthermore, a step-function Σ(τ) is defined as 1 for all t≥τ, and 0 otherwise.

## 4. Clustering Approach

Incorporating a second measurement method, namely RSS measurements, multiple adjacent nodes can be processed jointly to improve the positioning accuracy and mitigate outliers due to the DMC. We focus on a node of interest, $l^{\prime }$, at position $p_{l^{\prime }}$. We define a set of nodes L of size *N*, which incorporates all nodes *l*, which we want to include in our evaluation. This set L can be defined in various ways, but notably, we use RSS measurements and genie-aided methods to define the nodes within the set. The genie-aided method incorporates the N−1 nearest nodes (in geometrical sense) to $l^{\prime }$ and the node $l^{\prime }$ itself, where we use the ground-truth of all node positions $p_{l}$. The RSS-based method uses RSS measurements from the node $l^{\prime }$ to all other nodes *l*, and incorporates the N−1 nodes *l* with the largest RSSs and the node $l^{\prime }$ itself. For our purposes, it is assumed that the node positions $p_{l}$ are distributed around a mean cluster position $p_{c}\approx {\bar{\left\{p_{l}\right\}}}_{L}$. The index $l^{\prime }$ is omitted from here on to improve readability.

Assuming that measurements between different positions $p_{l}$ are independent, the joint likelihood for the set L equates to

(10) $$f\left(r|p_{c},\epsilon ,\eta ,\alpha \right)=\prod_{l\in L}\int_{p_{l}\in L}f_{l}\left(r_{l}|p_{l},\epsilon_{l},\eta_{l},\alpha_{l}\right)f\left(p_{l}|p_{c}\right)dp_{l},$$

where $\alpha ={\left[\alpha_{l}^{T}\right]}^{T}$, $\epsilon ={\left[\epsilon_{l}^{T}\right]}^{T}$, and $\eta ={\left[\eta_{l}^{T}\right]}^{T}\;\forall l\in L$ are stacked versions of their respective counterparts and r is a stacked vector of ${\left[r^{T}\right]}^{T}$ with l∈L. The term

(11) $$f\left(p_{l}|p_{c}\right)=\delta \left(p_{l}-p_{c}-\Delta_{l}\right)\approx \delta \left(p_{l}-p_{c}\right),$$

models the displacement between $p_{l}$ and $p_{c}$ by $\Delta_{l}$. The approximation assumes that the term $f(p_{l}|p_{c})$ is negligible because all likelihoods $f_{l}\left(r_{l}|p_{l},\epsilon_{l},\eta_{l},\alpha_{l}\right)$ have wide main lobes in comparison to the offset distance $\Delta_{l}$ from the cluster position $p_{c}$. Thus, a simplified log-likelihood for estimation and analysis is proposed as

(12) $$\ln f(r|p_{c},\epsilon ,\eta ,\alpha )=\sum_{l\in L}\ln f_{l}\left(r_{l}|p_{c},\epsilon_{l},\eta_{l},\alpha_{l}\right).$$

For this factorized likelihood function $f(r|p_{c},\epsilon ,\eta ,\alpha )$, it can be shown that there exists an unbiased estimator for the mean cluster position, if the regularity condition

(13) $$E\left[\frac{\partial \ln f(r|p_{c},\epsilon ,\eta ,\alpha )}{\partial p_{c}}\right]=0$$

holds true [30], because the distribution of $p_{l}\forall l\in L$ around $p_{c}$ is assumed to be the zero-mean. Note that (13) defines implicitly the exact cluster position $p_{c}$. This is similar to [31], where this assumption is used for spatial antenna arrays.

For all other parameters, this has already been shown in the literature [12,16].

## 5. Cramér-Rao Lower Bound

In order to evaluate the accuracy of results, the CRLB is derived, which is a general bound for the achievable accuracy. The CRLB is the inverse of the Fisher information (FI), for which derivations are shown in the following Subsections.

### 5.1. Introduction

The general form of the Fisher information matrix (FIM) for a PDF of the form f(r∣ψ) is [30,32]

(14) $$J_{\psi }=E_{r|\psi }\left[\left(\frac{\partial }{\partial \psi }\ln f\left(r|\psi \right)\right){\left(\frac{\partial }{\partial \psi }\ln f\left(r|\psi \right)\right)}^{T}\right],$$

for which the CRLB of an unbiased estimator $\hat{\psi }$ of the parameter vector ψ is defined as

(15) $$E_{\psi }\left[\left(\hat{\psi }-\psi \right){\left(\hat{\psi }-\psi \right)}^{H}\right]⪰J_{\psi }^{-1},$$

where it should be noted that A⪰B indicates that A−B is a positive semi-definite matrix.

### 5.2. Derivation of the Position Error Bound (PEB)

To derive lower bounds on the error variance of position estimates, we first define a parameter vector $\psi_{l,m}={\left[\phi_{l,m},\vartheta_{l,m},\tau_{l,m},R\alpha_{l,m},I\alpha_{l,m}\right]}^{T}$ with an azimuth angle from anchor *m* to node *l* of $\phi_{l,m}=\mathrm{atan}2\left(y_{l}-y_{m},x_{l}-x_{m}\right)$, with elevation angle $\vartheta_{l,m}=\mathrm{atan}2\left(z_{l}-z_{m},\sqrt{{\left(x_{l}-x_{m}\right)}^{2}+{\left(y_{l}-y_{m}\right)}^{2}}\right)$, and delay $\tau_{l,m}=\tau_{m}\left(p_{l}\right)+\epsilon_{l}$.

The equivalent Fisher information matrix (EFIM) [33] on the delay $\tau_{l,m}$ and angle measurements $\phi_{l,m}$ and $\vartheta_{l,m}$ acquired by anchor *m* on each node *l* is then given as

(16) $$J_{\psi_{l,m}}=\left[\begin{array}{ccc} J_{\phi_{l,m}} & 0 & 0\\ 0 & J_{\vartheta_{l,m}} & 0\\ 0 & 0 & J_{\tau_{l,m}} \end{array}\right],$$

where the diagonal elements $J_{\phi_{l,m}}$, $J_{\vartheta_{l,m}}$, and $J_{\tau_{l,m}}$ account for the information with respect to the different parameters. A derivation of this EFIM is given in Appendix A. Furthermore, we introduce the Jacobian matrix $P_{m}\left(p_{l}\right)$ for transforming spherical to Cartesian coordinates, which is defined as

(17) $$\begin{array}{c} P_{m}\left(p_{l}\right)=\left[\begin{array}{ccc} \frac{\partial x_{l}}{\partial \phi_{l,m}} & \frac{\partial x_{l}}{\partial \vartheta_{l,m}} & \frac{\partial x_{l}}{\partial \tau_{l,m}}\\ \frac{\partial y_{l}}{\partial \phi_{l,m}} & \frac{\partial y_{l}}{\partial \vartheta_{l,m}} & \frac{\partial y_{l}}{\partial \tau_{l,m}}\\ \frac{\partial z_{l}}{\partial \phi_{l,m}} & \frac{\partial z_{l}}{\partial \vartheta_{l,m}} & \frac{\partial z_{l}}{\partial \tau_{l,m}} \end{array}\right]\\ =\left[\begin{array}{ccc} \frac{\sin\phi_{l,m}\sin\vartheta_{l,m}}{\tau_{l,m}c} & \frac{\cos\phi_{l,m}\cos\vartheta_{l,m}}{\tau_{l,m}c} & \frac{\sin\phi_{l,m}\cos\vartheta_{l,m}}{c}\\ \frac{\sin\phi_{l,m}\cos\vartheta_{l,m}}{\tau_{l,m}c} & \frac{\cos\phi_{l,m}\sin\vartheta_{l,m}}{\tau_{l,m}c} & \frac{\sin\phi_{l,m}\sin\vartheta_{l,m}}{c}\\ 0 & -\frac{\sin\phi_{l,m}}{\tau_{l,m}c} & \frac{\cos\phi_{l,m}}{c} \end{array}\right]\in R^{3\times 3}, \end{array}$$

for node l∈L at position $p_{l}={\left[x_{l},y_{l},z_{l}\right]}^{T}$. Note that *c* is the speed of light.

Assuming independent noise for all nodes l∈L, and knowledge of the displacement $\Delta_{l}=p_{l}-p_{c}$ between the node positions $p_{l}$ and the cluster position $p_{c}$, the Cartesian EFIM for the cluster is expressed by the sum of the node EFIMs,

(18) $$J_{p_{c}}^{\left(m\right)}=\sum_{l\in L}P_{m}\left(p_{l}\right)J_{\psi_{l,m}}{P_{m}\left(p_{l}\right)}^{T}=P_{m}\left(p_{c}\right)J_{\psi_{m}^{\left(c\right)}}P_{m}{\left(p_{c}\right)}^{T}.$$

The right-hand side of this expression transforms the sum-information in Cartesian coordinates back to range and angle measurements. This yields a (non-diagonal) EFIM $J_{\psi_{m}^{\left(c\right)}}$ for the cluster center as

(19) $$J_{\psi_{m}^{\left(c\right)}}=\sum_{l\in L}P_{m}{\left(p_{c}\right)}^{-1}P_{m}\left(p_{l}\right)J_{\psi_{l,m}}{\left(P_{m}{\left(p_{c}\right)}^{-1}P_{m}\left(p_{l}\right)\right)}^{T},$$

where the off-diagonal elements describe some transformation of angle information to delay-information and vice versa. We thus argue that a diagonal EFIM, written as a sum of all delay and angle terms, serves as an upper bound on the delay and angle information for the cluster at $p_{c}$, if the position offsets $\Delta_{l}$ are unknown, i.e., we have

(20) $${\tilde{J}}_{\psi_{m}^{\left(c\right)}}=\sum_{l\in L}J_{\psi_{l,m}}$$

as an upper bound on the sum-information from all the delay and angle measurements for all nodes of the cluster and the corresponding bound on the position information,

(21) $${\tilde{J}}_{p_{c}}^{\left(m\right)}=P_{m}\left(p_{c}\right){\tilde{J}}_{\psi_{m}^{\left(c\right)}}P_{m}{\left(p_{c}\right)}^{T}.$$

From this, we define a multi-anchor EFIM for the cluster position $p_{c}$, which is decomposed into three components corresponding to the delay and angle terms as

(22) $$\begin{array}{cc} J_{p_{c}}=\sum_{m=1}^{M} & {\tilde{J}}_{p_{c}}^{\left(m\right)} \end{array}$$

(23) $$\begin{array}{cc} \approx \sum_{m=1}^{M}\; & [\frac{8\pi^{2}}{d_{m}^{2}}D_{\lambda }^{2}\left(\phi_{m}\right)\;KL\;{\mathrm{SINR}}_{m}R_{r}\left(\phi_{m}+\frac{\pi }{2},\vartheta_{m}\right) \end{array}$$

(24) $$\begin{array}{cc} + & \frac{8\pi^{2}}{d_{m}^{2}}D_{\lambda }^{2}\left(\vartheta_{m}\right)\;KL\;{\mathrm{SINR}}_{m}R_{r}\left(\phi_{m},\vartheta_{m}+\frac{\pi }{2}\right) \end{array}$$

(25) $$\begin{array}{cc} + & \Lambda \frac{8\pi^{2}}{c^{2}}\beta^{2}\;KL\;{\left[{\tilde{\mathrm{SINR}}}_{\tau }\right]}_{m}R_{r}\left(\phi_{m},\vartheta_{m}\right)], \end{array}$$

using the results from Appendix B and Appendix C. Here, $d_{m}$, $\phi_{m}$, and $\vartheta_{m}$ are the distances between the array centers and the cluster center and the corresponding angle parameters. $D_{\lambda }^{2}\left(\phi_{m}\right)$ and $D_{\lambda }^{2}\left(\vartheta_{m}\right)$ are the array apertures in azimuth and elevation, $\beta^{2}$ is the mean-squared signal bandwidth, and ${\mathrm{SINR}}_{m}$ and ${\left[{\tilde{\mathrm{SINR}}}_{\tau }\right]}_{m}$ account for the interference by the dense multipath (DM). The factor KL quantifies the number of antennas per anchor, as well as the number of nodes in the cluster, which are interpreted as a boost in SINR, i.e., a suppression of the influence of the DM. Finally, the matrices $R_{r}\left(\phi_{m},\vartheta_{m}\right)$ are so-called ranging direction matrixs (RDMs) [33] defined as

(26) $$R_{r}\left(\phi_{m},\vartheta_{m}\right)=e\left(\phi_{m},\vartheta_{m}\right)e^{T}\left(\phi_{m},\vartheta_{m}\right)$$

where $e(\phi_{m},\vartheta_{m})$ is a unit vector pointing from array *m* in the direction of node *l*.

This position error bound (PEB) (see (22)) also takes the correction factor Λ into account for the clustering, dissimilar to other related work only showing results for single nodes. It accounts for the spread of delays $\tau_{m}\left(p_{l}\right)$ for l∈L around $\tau_{m}\left(p_{c}\right)$, which leads to an apparent loss of bandwidth as derived in Appendix B.

With this, we can define the PEB for multiple anchors *m* and multiple nodes l∈L as

(27) $$P_{c}=\sqrt{\mathrm{tr}\{J_{p_{c}}^{-1}\}}.$$

### 5.3. Biased Lower Bound

In the previous subsection, we derived the PEB for the cluster position $p_{c}$. For real-world applications, the cluster position would be mostly of no importance, but it can be assumed that the cluster position is within a vicinity of the position of the node $l^{\prime }$, which has been used to define the cluster. Equality holds true for scenarios where all nodes $l\in L\setminus \{l^{\prime }\}$ are distributed in such a way that the cluster position $p_{c}=p_{l^{\prime }}$. In real world scenarios, this assumption often does not hold; thus, we propose a biased lower bound for the node $l^{\prime }$

(28) $$P_{l^{\prime }}=P_{c}+∥p_{c}-p_{l^{\prime }}∥.$$

This biased lower bound has the advantage of easier representation of relevant errors, and comparability with the root-mean-square error (RMSE) of the first node position ${\hat{p}}_{l^{\prime }}$.

## 6. Numeric Evaluation

Note that in this section, if there is a discussion about the cluster position $p_{c}$ or the position of the first node in a cluster $p_{l^{\prime }}$, it is always assumed to be for every possible set L for every node l∈L, meaning that this evaluation is conducted for every possible cluster in a scenario. To validate the CRLB and biased bound for $p_{l^{\prime }}$, we evaluated four simulation scenarios, two of which represent a realistic indoor scenario. Lastly, an evaluation of measurement data was conducted, where the scenario corresponded to our simulations. For the evaluation, only the AWGN case was considered, and all estimations were conducted in practice via a particle evaluation of the joint-likelihood $f(r|p_{c},\eta ,\alpha )$ [34], described in (12), by

(29) $${\hat{p}}_{c}=\underset{p_{c},\epsilon_{l}\forall l\in L}{\arg\max}\;f\left(r|p_{c},\epsilon ,\eta ,\alpha \right).$$

For the parameter α, a least-squares solution can be found as

(30) $${\hat{\alpha }}_{l,m}\left(p_{c}\right)={\left[s_{l,m}{\left(p_{c},\epsilon_{l}\right)}^{H}s_{l,m}\left(p_{c},\epsilon_{l}\right)\right]}^{-1}s_{l,m}{\left(p_{c},\epsilon_{l}\right)}^{H}r_{l,m},$$

calculated for every element $\alpha_{l,m}$. The parameter ϵ had to be estimated by the particle filter. This particle filter allowed us to estimate the position of a cluster with size N=40 on a typical workstation in under 30 s of time, and for small cluster sizes, the computation time decreases linearly.

As the estimations of DMC parameters are omitted, the state of this joint-likelihood can be represented by a parameter vector $\nu ={\left[x_{c},y_{c},z_{c},\epsilon_{1},\cdots ,\epsilon_{N_{l}}\right]}^{T}$, where $\epsilon_{l}$ represents the transmit time of each node in the set L, and $N_{l}$ represents the number of elements in the set L. For the initialization of the parameters ${\left[x_{c},y_{c}\right]}^{T}$, a support over the simulated room was chosen. For the height $z_{c}$, the support was chosen to be in the range [−6 m,6 m] for scenarios with no shelf simulation, and [0 m,2 m] for scenarios with shelf simulation. For the measurement scenario, the particles were initialized within the aisles only. Transmit times $\epsilon_{l}$ were initialized on an interval of $\left[\frac{0\;m}{c},\frac{150\;m}{c}\right]$, which represents all transmit times in simulated and measured scenarios within a reasonable margin. Note that *c* again represents the speed of light.

Each particle $\nu_{p}$ is initialized as one realization of the parameter vector ν, meaning that each particle represents a cluster position and unknown transmit times of all nodes within the cluster. The likelihood is then evaluated for each and every particle.

All evaluations are conducted with 1000 particles, each of which represents one state $\nu_{p}$. This was a reasonable trade-off between estimation time and accuracy. A three-step approach was chosen. After an initalization step, particles were resampled twice from the computed likelihoods. The first resampling step was completed by resampling from the computed normalized log-likelihood and adding additional i.i.d. Gaussian noise to every resampled parameter. This noise was chosen as an i.i.d. Gaussian process described by a mean of 0 m and a standard deviation of 0.5 m for parameters ${\left[x_{c},y_{c},z_{c}\right]}^{T}$ and $0.5\;m\frac{1}{c}$ for parameters $\epsilon_{l}$. The additional noise helps mitigating particle deprivation, where all particles are resampled from a single previous particle with an exceptionally large likelihood compared with all other particles. This first resampling step was chosen to better ensure resampling from modes which were underrepresented in the initialization step, but avoiding a much larger number of particles beforehand. This strategy leads to a very coarse maximum for the particles, and thus a second resampling step is necessary. There, we used a classical strategy where the resampling was conducted directly from the estimated normalized likelihood, once again adding the same i.i.d. noise. This resampling focused more on the dominant mode of the resulting distribution, and thus can be seen as a refinement in the vicinity of the dominant mode. This two-step resampling strategy allows us to use significantly fewer particles for our estimation problem. By only using the second resampling step, we needed a factor of at least 20 times the number of particles for comparable accuracy, which leads to a proportional increase of calculation time by the same factor. It should be noted that this implementation is capable of calculating positions in real time, as the number of operations to be processed is fixed by the number of particles.

Note that the simulations and measurements used six antenna arrays with two antennas each. This is not a limitation of the algorithm. Arbitrary antenna configurations can be used, but the configuration in the simulations was chosen for easier comparison to the measurement scenario.

### 6.1. Cluster in Single Position

For a first evaluation, we show that the PEB for the cluster position $p_{c}$ holds true. To achieve this, we simulated a scenario with following parameters.

**Figure 2:**
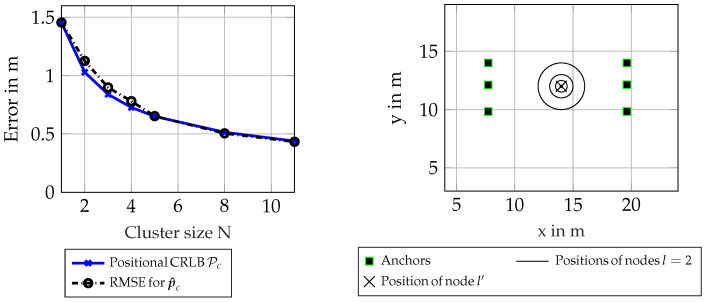
Scenario 6.1 and 6.2. Results for synthetic scenario in single position with random clusters and schematic plot for clusters of size 1 and size 2 with variable distances.

For placement of the antenna arrays, see Figure 2. All antenna-arrays are shown as squares, and the positions of all nodes *l* are shown as a black cross. The arrays are all oriented such that they are spaced in the direction of the y-axis; therefore, the positioning performance improves with the aperture of the arrays. The nodes are placed at $p_{c}={\left[14\;m,\;12\;m,\;1\;m\right]}^{T}$. For this scenario, 1100 realizations of a channel incorporating DMC was chosen, with an SNR at 1 m of $\frac{\alpha_{1}^{2}}{\sigma_{l,m,k}^{2}}=25\;\mathrm{dB}$, where $\alpha_{1\;m}$ is the equivalent amplitude of the normalized signal at 1 m. The cluster-size *N* was varied, where the first node was always one unique node from all 1110 simulated nodes, and every other node in the set was chosen randomly from all other remaining nodes.

The signal amplitude was then scaled according to the Friis equation, leading to

(31) $$\alpha_{l,m}=\alpha_{1\;m}\left(\frac{\lambda_{c}}{4\pi d_{l,m}}\right),$$

where $\lambda_{c}=\frac{1}{f_{c}}$ and $d_{l,m}$ is the distance between node *l* and anchor *m*. The parameter $\Omega_{1}$ of the DMC at 1 m was drawn from an i.i.d. Gaussian random process, and scaled according to the Friis Equation [35] resulting in

(32) $${\left[\Omega_{1}\right]}_{l,m}∼N\left(\mu =0\;\mathrm{dB},\sigma^{2}=2.16\;\mathrm{dB}\times {\left(\frac{\lambda_{c}}{4\pi d_{l,m}}\right)}^{2}\right),$$

where $N(\mu ,\sigma^{2})$ is a random i.i.d Gaussian process. This describes one realization of the random variable for the parameter $\Omega_{1}$. The parameters for the fall and rise time of the DMC were chosen as fixed values being $\gamma_{r}=5\;\mathrm{ns}$ and $\gamma_{f}=20\;\mathrm{ns}$, which are typical values for an indoor scenario [29,36].

Figure 2 shows that the CRLB can be attained for this scenario, as the curve showing the RMSE for ${\hat{p}}_{c}$ is almost identical to the theoretical bound. A small offset can be seen for small cluster sizes (i.e., N in the range of 2 to 4), which can be attributed to the particle-based estimation not being perfect. This could be mitigated by using more particles. Note that the bias $∥p_{c}-p_{l^{\prime }}∥$ and correction factor Λ were both zero for all different cluster sizes in this scenario, and therefore, the equivalent Fisher information (EFI) could be directly summed up, meaning the CRLB scaled with $\frac{1}{\sqrt{N}}$, which can be interpreted as using different realizations from the same node.

### 6.2. Double-Node Clusters with Variable Distance

For this scenario, the same settings as in the previous section were used regarding antenna placement and signal parameters, see Table 1. All other parameters of the signal are also defined the same as in Section 6.1. In this scenario, 1110 pairs of nodes were simulated, where for every node $l^{\prime }$ a new realization of the channel was drawn, with position $p_{l^{\prime }}$ being the same as in the previous scenario, and for the corresponding node l=2, another realization of the channel was drawn at a position $p_{2}=p_{l^{\prime }}+{\left[d_{r}\cos\phi_{r},d_{r}\sin\phi_{r},0\right]}^{T}$, where the distance $d_{r}$ was evaluated at four distances from 0.1 m to 2 m, and the angle $\phi_{r}$ was drawn from a uniform random distribution with $\phi_{r}∼\left[0,2\pi \right)$. This is visualized as concentric circles in Figure 2.

**Figure 3:**
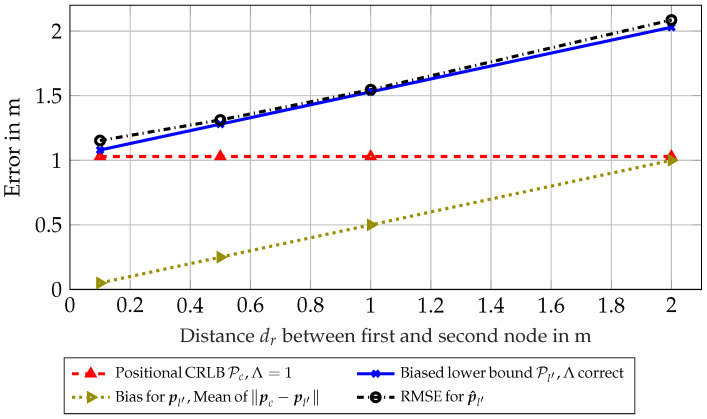
Scenario 6.2: Results for synthetic scenario for clusters of size N = 2 with fixed distance in a circle around node $l^{\prime }$.

As can be seen in Figure 3, the PEB $P_{c}$, omitting the correction factor Λ, is constant over distance, which is not representative for the RMSE for the estimated position ${\hat{p}}_{l^{\prime }}$ of the first node $l^{\prime }$. Correcting for the mean distance $∥p_{c}-p_{l^{\prime }}∥$, one can see that the performance of the estimator is attaining the biased lower bound $P_{l^{\prime }}$. Note that this biased lower bound is not only a linear offset due to geometric distance, but also incorporates a small information loss by the introduction of the evaluated correction factor Λ. This simulation shows that the introduction of a lower bound incorporating a bias between cluster center $p_{c}$ and the position of the first node $p_{l^{\prime }}$ can be replicated in an evaluation of the according RMSE.

### 6.3. Simulated Scenario with Genie-Aided Clusters

**Figure 4:**
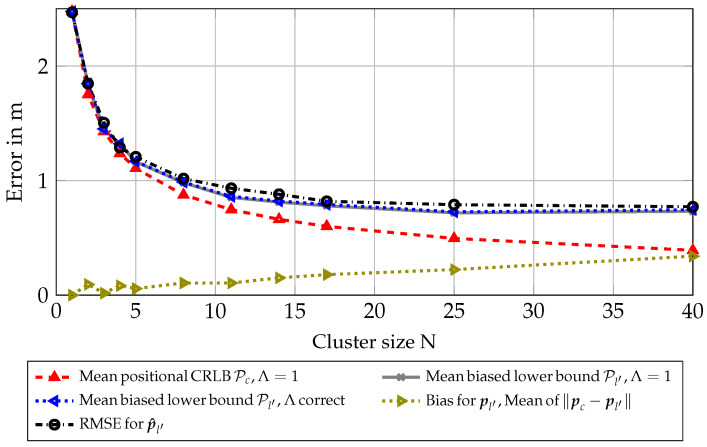
Scenario 6.3: Results for synthetic scenario with genie-aided clusters (minimum distance to node 1).

To further test our algorithm in a more realistic scenario, we simulated a room where 1110 nodes are placed on shelves at different heights and positions in a room, for reference see Figure 1. The signal parameters were again chosen according to Table 1, and parameters $\Omega_{1}$ and $\alpha_{l,m}$ were again scaled according to Equations (31) and (32). For each intersection with a shelf of a ray casted from antenna array position $p_{m}$ to a node position $p_{l}$, distributions of parameters $\Omega_{1}$ and $\alpha_{l,m}$ are changed according to Table 2. These values were chosen to represent empirical measurements for this type of shelf in previous work [37]. Sets L for clusters were chosen to minimize geometric cluster sizes, where nearest nodes from $l^{\prime }$ were selected, assuming side information provided by a genie. This was conducted to show a perfect scenario, minimizing other possible unknown effects to the estimation, and incorporates no model for the RSS, only geometric distances. As can be seen in Figure 4, the RMSE almost attains the biased lower bound in this scenario. This shows that for a perfect selection of nearest nodes, the estimation of the position $p_{c}$ can also be seen as a good estimate for the position $p_{l^{\prime }}$ of the first node in a cluster, improving accuracy significantly with bigger cluster sizes. As sizes of the clusters are relatively small, the correction factor Λ is almost negligible for all cluster sizes in this scenario. This can be seen in the lines for the biased lower bound with Λ=1 and the biased lower bound with Λ correct being almost identical.

**Figure 5:**
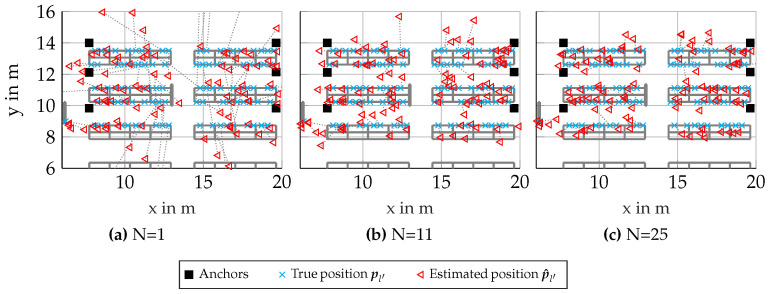
Scenario 6.3: Floorplans with results for synthetic scenario with genie-aided clusters (showing only every 10th processed node for better visibility).

In Figure 5a–c the floorplans of the scenario can be seen, where red triangles show the estimated position and cyan crosses show the true position for every 10th simulated node, omitting many measurements for better visibility. The true and estimated positions are connected by a dotted gray line. Similar to Figure 1, anchors are again shown as black squares. In Figure 5a, many estimated node positions are outliers. This is due to the multi-modality of the likelihood for single nodes leading to false positions in the likelihood being dominant. This can be mitigated mostly by larger cluster sizes N, which can be seen in Figure 5b,c, where the position errors improve with larger cluster size N.

### 6.4. Simulated Scenario with RSS-Based Clusters

To further show that the clustering approach works for realistic scenarios, the same simulation as in Section 6.3 was conducted, but clusters were defined according to real RSS measurements in a corresponding measurement scenario. All RSS measurements between every pair of nodes were known. Clusters were then defined by sorting the RSS values from the $l^{\prime }$th node to every other node in descending order, achieving the now measured sets L for every node $l^{\prime }$. As these measurements are noisy and incorporate channel parameters, geometric cluster sizes are bigger and often biased, for example, in the direction of an aisle, compared to the genie-aided clusters in the previous subsection.

**Figure 6:**
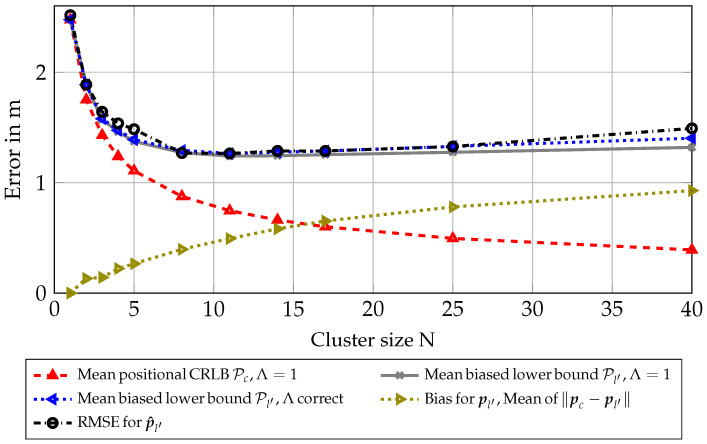
Scenario 6.4: Results for synthetic scenario with RSS-based clusters.

Figure 6 shows the same results as in Figure 4, but with clusters based on RSS measurements. The mean bias for $p_{l^{\prime }}$ is now significantly larger, leading to a diminished performance for bigger cluster sizes *N*. Still, the RMSE mostly attains the biased lower bound. It should be noted that the correction factor Λ is now showing a significant offset for larger clusters with the RMSE following the resulting offset. It can be seen that the effects of the bias for $p_{l^{\prime }}$ and the correction factor Λ dominate over the classical CRLB for large cluster sizes *N*, meaning that there is an optimum for the cluster size depending on the scenario.

**Figure 7:**
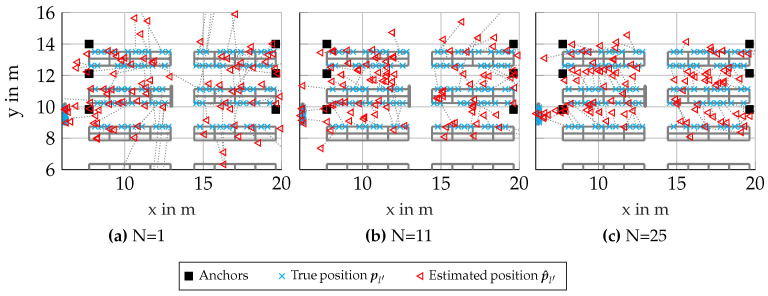
Scenario 6.4: Floorplans with results for synthetic scenario with RSS-based clusters (showing only every 10th processed node for better visibility).

Figure 7 again shows a map of the environment as previously seen in Figure 5a. Note that the results for single nodes differ, as these evaluations were conducted with different realizations for the channel. Now, for larger cluster sizes, as seen in Figure 7b,c, it can be seen that the estimated positions ${\hat{p}}_{l^{\prime }}$ are increasingly shifted into the aisles. This effect is attributed to the RSS measurements showing smaller values when the propagation path is through a shelf, which is an expected behavior of the channel, biasing our clusters towards the centers of the aisles. As seen in Figure 6, the RMSE is lower for clusters of size N=11 than for clusters of size N=25. This can be seen as smaller absolute errors in Figure 7b than in Figure 7c, but for some scenarios, it may be preferable to achieve a slightly larger absolute positioning error in exchange for a more accurate classification of positions to the right aisle and/or shelf.

### 6.5. Experimental Validation

**Figure 8:**
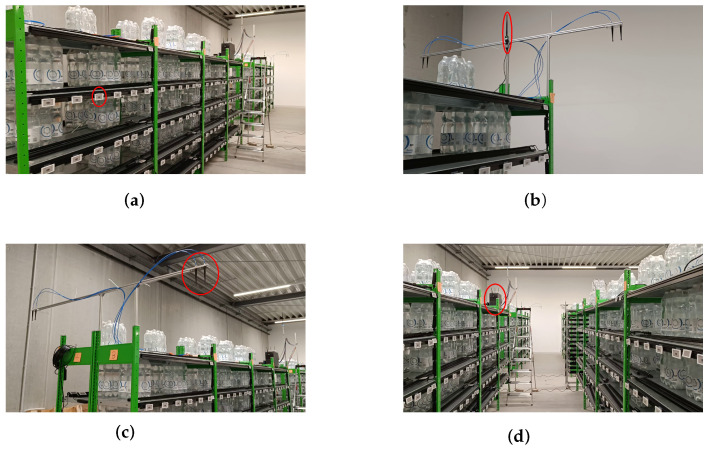
Scenario 6.4 and 6.5: Pictures of the measurement setup and room. (**a**) Red ellipse marking one agent node. (**b**) Red ellipse marking the access point antenna for controlling the agent nodes. (**c**) Red ellipse marking one linear 2-antenna array. (**d**) Red ellipse marking the PC for measurement processing.

Figure 8 shows parts of the measurement setup and room. Here, one can see the placement of nodes (in this case, electronic shelf labels) mounted on industrial shelves, similar to those found in retail stores. The antennas seen in the picture correspond to the two upper-left positions for anchors seen in Figure 1. The nodes allowed for cooperative RSS measurements between each pair of nodes by using a proprietary protocol and transmission in the ISM band at 2.4 GHz. The WB measurements were conducted according to a protocol described in detail in [4,38].

To further validate results, we used WB measurement data from a scenario corresponding to our simulations. The geometry and number of anchors were the same as in the simulation scenarios in the previous two subsections; parameters from Table 1 still apply. Corresponding RSS measurement data were also retrieved. These measurements were for all 1100 nodes in a timeframe of approximately 24 h, where most time was used for transmission of the RSS measurement data to the infrastructure. In comparison, a single WB measurement took less than one minute with additional post-processing. Both genie-aided and RSS data-based clustering approaches were evaluated. As we wanted these evaluations to show the optimal performance we could achieve in such a scenario, we further incorporated prior knowledge of the agent positions, restricting all particles to areas within aisles (see Figure 1 for reference). This allows us to improve performance by incorporating knowledge about the room.

**Figure 9:**
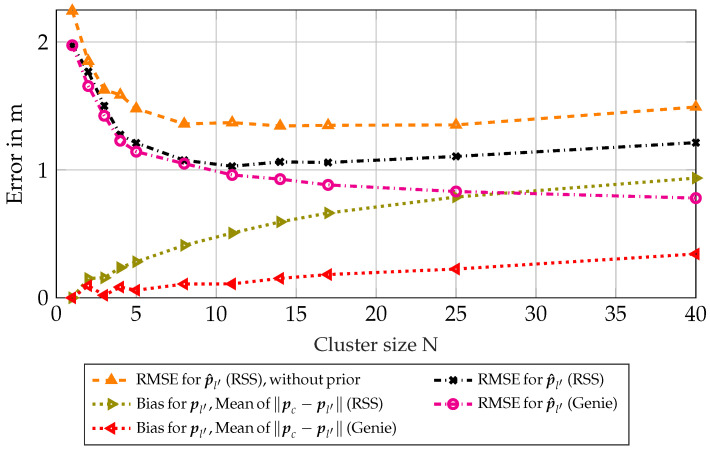
Scenario 6.5: Results for measurement scenario.

Figure 9 shows the measurement scenario. Note that the CRLB and biased lower bound are not shown here, as an accurate estimation of these bounds requires appropriate channel parameters, which cannot be extracted from our measurements well enough. Therefore, one can see that the performance of the clustering approaches corresponds to the two simulation scenarios seen in Section 6.3 and Section 6.4. It can also be seen that for the genie-aided clustering, the performance increased for all cluster sizes in this evaluation, though we expect the performance to worsen for even larger cluster sizes, as the bias term dominates for large clusters. The evaluation using RSS measurement data again shows an ideal cluster size for the RMSE of N=11, meaning that for real-world scenarios, using the RSS-based clustering approach there is no need for more computationally intensive, larger cluster sizes. For reference, the RMSE is also plotted for an evaluation using RSS measurements, which does not incorporate a prior in the aisles. This evaluation was conducted with the same number of particles, as these proved to be sufficient. Here, we see that the estimation performance was worse over all cluster sizes, justifying the initialization within aisles. As a side note, an evaluation with non-fixed cluster sizes, where clusters were determined by RSS-thresholds was also conducted. Over a wide range of thresholds, this did not improve the results.

**Figure 10:**
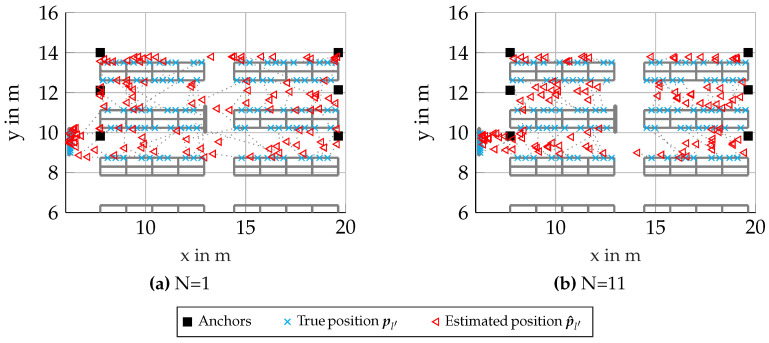
Scenario 6.5: Floorplans with results from measurements with RSS-based clusters (showing only every 10th processed node for better visibility).

As seen in Figure 10a, comparable to the synthetic scenarios, the estimation errors for non-clustered processing were rather large. Some effects of the geometry were more pronounced, which can be seen, for example, in the left-most nodes at x≈6 m,y≈9 m, being estimated very accurately in comparison with most other nodes. As these nodes are in the direct vicinity of an antenna array, there is a pronounced LoS-component and a good SNR for WB measurements. Furthermore, the effects of the initialization within possible aisles already leads to better results for the non-clustered case.

Looking at Figure 10b, we again see a significant performance improvement for the clustering algorithm using RSS-based clusters. It is worth noting that, again, the estimated positions ${\hat{p}}_{l^{\prime }}$ are increasingly shifted to within centers of aisles, which again, can contribute to RSS measurements giving the stronger indications for links with LoS conditions, which often happen to be on the opposite side of aisles for our scenario.

**Figure 11:**
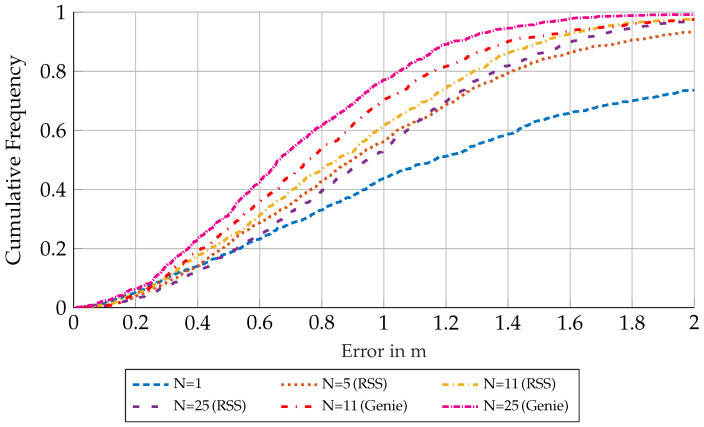
Scenario 6.5: CF plot and excerpt for different cluster sizes and cluster selection strategies.

Figure 11 shows a plot for the cumulative frequency (CF) of the error $∥{\hat{p}}_{c}-p_{l^{\prime }}∥$ for both genie-aided and RSS-based clustering approaches. It can be seen that especially outliers can be minimized by the clustering approaches compared to the non-clustered (N=1) case. It should be noted that the genie-aided approach clearly outperforms the RSS-based method, but for the minimization of outliers (>2 m), both methods are almost equal when using cluster sizes N≥11. Furthermore, one can see that the performance of the RSS-based clusters is worse for very small errors (<0.2 m) than for a non-clustered approach. This can be attributed to the bias-term being dominant, as RSS-measurements to nodes on the other side of an aisle were often dominating, even in small cluster sizes.

## 7. Conclusions

This paper investigates an indoor position system that is capable of fusing WB ToF measurements and cooperative RSS measurements from a cluster of nodes located in close proximity to one another. We derive a lower bound on the position error for this setup to understand the scaling behavior and performance limits. We also formulate an approximated maximum likelihood algorithm for the setup and analyze the performance with synthetic data and real-world measurement data.

Our proposed approach demonstrates a performance gain of one node for a non-clustered approach being around 2 m to the performance of an RSS-clustered approach being around 1 m in our measurement scenario. Synthetic scenarios show that our RMSE can approach the derived biased lower bound, incorporating a correction factor that accounts for the precise scaling of delay information in the case of clustering.

Furthermore, an efficient implementation has been developed for our algorithm, based on an iterative implementation of a maximum likelihood estimator. Overall, we were able to show that the introduced approach mitigates mutual problems of both measurement methods, improving localization performance by incorporating information that can be processed jointly.

Results for the fusion of multiple measurement methods—like the ones evaluated—show that this is a promising field for future research, and further work should incorporate other measurement methods and additional information to improve upon the principle findings of this paper. Future work for the presented method will focus on a fully Bayesian implementation supporting joint cooperative positioning based on both measurement types, directly incorporating the information content of RSS measurements into a joint algorithm.

## Figures and Tables

**Table 1:** Settings for simulation.

$f_{c}$	β	*M*	*K*	Antenna Spacing
2.44 GHz	75.3 MHz	6	2	6 cm

**Table 2:** Change of parameters.

Intersections	0	1	2	3
μ of $\Omega_{1}$	−20 dB	−10 dB	0 dB	10 dB
$\sigma^{2}$ of $\Omega_{1}$	2.16 dB	5.30 dB	7.15 dB	6.40 dB
$\frac{\alpha_{l,m}^{2}}{\sigma_{l,m,k}^{2}}$ at 1 m	25 dB	21.42 dB	19.46 dB	19.56 dB

## Data Availability

For non-commercial use in research, data sets are available on request.

## Abbreviations

The following abbreviations are used in this manuscript:

AEB	angulation error bound
AoA	angle of arrival
AP	access point
AWGN	additive white Gaussian noise
CF	cumulative frequency
CRLB	Cramér-Rao lower bound
DM	dense multipath
DMC	dense multipath component
DPS	delay power spectrum
FI	Fisher information
EFI	equivalent Fisher information
EFIM	equivalent Fisher information matrix
FIM	Fisher information matrix
IoT	internet of things
ISM	industrial, scientific, and medical
LoS	line-of-sight
MCS	Monte-Carlo simulation
PDF	probability density function
PEB	position error bound
PSD	power spectral density
RDM	ranging direction matrix
REB	ranging error bound
RFID	radio frequency identification
RMSE	root-mean-square error
RSS	received signal strength
RSSI	received signal strength indicator
SNR	signal-to-noise ratio
SINR	signal-to-interference-plus-noise-ratio
TDoA	time difference of arrival
ToA	time of arrival
ToF	time of flight
UWB	ultra-wideband
WB	wideband

## Appendix A. Derivation of EFIM

For ease of notation, indices *l* and *m* are omitted here without loss of generality, but these derivations apply for a single anchor *m* and node *l*. To derive position error bounds, we first derive an FIM for a spherical parameter vector $\psi ={\left[\phi ,\vartheta ,\tau ,R\alpha ,I\alpha \right]}^{T}$ with nuissance parameter α∈C, as

(A1) $$J_{\psi }=\left[\begin{array}{cc} A & B\\ B^{T} & D \end{array}\right]\in R^{5\times 5}.$$

The block matrices are defined similar to [16] as

(A2) $$\begin{array}{cc} A & =\left[\begin{array}{ccc} J_{\phi \phi } & 0 & 0\\ 0 & J_{\vartheta \vartheta } & 0\\ 0 & 0 & J_{\tau \tau } \end{array}\right]\\ B & =\left[\begin{array}{cc} 0 & 0\\ 0 & 0\\ J_{\tau R\alpha } & J_{\tau I\alpha } \end{array}\right]\\ D & =\left[\begin{array}{cc} J_{R\alpha R\alpha } & 0\\ 0 & J_{I\alpha I\alpha } \end{array}\right]. \end{array}$$

The FIs between the parameters in the according subscript are denoted as $J_{\phi \phi }$, $J_{\vartheta \vartheta }$,$J_{\tau \tau }$, $J_{\tau R\alpha }$, $J_{\tau I\alpha }$, $J_{R\alpha R\alpha }$, $J_{I\alpha I\alpha }$. Note that the transmit time $\epsilon_{l}$ is also omitted, as for TDoA positioning an unknown transmit time has negligible influence [39], given an appropriate geometry of anchor nodes. To gain further insight, we use the EFIM ${\left[J_{\psi^{\prime }}\right]}_{3\times 3}^{-1}$ for the parameter vector $\psi^{\prime }={\left[\phi ,\vartheta ,\tau \right]}^{T}$ by using the Schur complement on (A1)

(A3) $${\left[{J_{\psi^{\prime }}}^{-1}\right]}_{3\times 3}={\left(A-BD^{-1}B^{T}\right)}^{-1}={\left[\begin{array}{ccc} J_{\phi } & 0 & 0\\ 0 & J_{\vartheta } & 0\\ 0 & 0 & J_{\tau } \end{array}\right]}^{-1},$$

where $J_{\phi }$, $J_{\vartheta }$, and $J_{\tau }$ are the respective FI terms for the parameters. Due to the structure of the block matrices A, B, and D, the EFIM ${\left[J_{\psi^{\prime }}^{-1}\right]}_{3\times 3}$ is a diagonal matrix, illustrating that range and angle information components are independent. To derive a CRLB for positioning, a transformation of the EFIM from the spherical parameter vector to a cartesian parameter vector $p={\left[x,y,z\right]}^{T}$ is necessary. For this, the corresponding FIM for the parameter vector p can be computed by

(A4) $$J_{p}=TJ_{\psi^{\prime }}T^{T},$$

with T being the Jacobian matrix for transformation of spherical to cartesian coordinates incorporating partial derivatives of p with respect to $\psi^{\prime }$

(A5) $$T=\frac{{\partial p}^{T}}{\partial \psi^{\prime }}=\left[\begin{array}{ccc} \frac{\partial x}{\partial \phi } & \frac{\partial x}{\partial \vartheta } & \frac{\partial x}{\partial \tau }\\ \frac{\partial y}{\partial \phi } & \frac{\partial y}{\partial \vartheta } & \frac{\partial y}{\partial \tau }\\ \frac{\partial z}{\partial \phi } & \frac{\partial z}{\partial \vartheta } & \frac{\partial z}{\partial \tau } \end{array}\right]=\left[\begin{array}{ccc} \frac{\sin\phi \sin\vartheta }{\tau c} & \frac{\cos\phi \cos\vartheta }{\tau c} & \frac{\sin\phi \cos\vartheta_{l,m}}{c}\\ \frac{\sin\phi \cos\vartheta }{\tau c} & \frac{\cos\phi \sin\vartheta }{\tau c} & \frac{\sin\phi \sin\vartheta }{c}\\ 0 & -\frac{\sin\phi }{\tau c} & \frac{\cos\phi }{c} \end{array}\right]\in R^{3\times 3},$$

where *c* denotes the speed of light.

## Appendix B. Derivation of the Ranging Error Bound (REB) and Correction Factor Λ

### Appendix B.1. Ranging Error Bound

The ranging error bound (REB) is derived from the likelihood function (7). To account for the DMC, an signal-to-interference-plus-noise-ratio (SINR) is introduced, which quantifies the reduction of the SNR due to the interfering DM [16,39]. With this, we can define the EFI (which is the equivalent information neglecting nuisance parameters) for the delay $\tau_{l,m}$ for a single node as [16,39]

(A6) $$\begin{array}{c} J_{\tau_{l,m}}=8\pi^{2}\beta^{2}{\tilde{\mathrm{SINR}}}_{\tau_{m}}K, \end{array}$$

where $\beta^{2}$ is the mean-squared bandwidth [40], which is defined as $\beta^{2}=∥\dot{s}{∥}^{2}/\left(4\pi^{2}{∥s∥}^{2}\right)=\int_{f}f^{2}{\left|S\left(f\right)\right|}^{2}df$ for the normalized pulse ${∥s∥}^{2}T_{s}=1$, $\dot{s}$ being the derivative of the sampled pulse with respect to the delay τ, and *f* is the frequency in the Fourier domain, ${\left[{\tilde{\mathrm{SINR}}}_{\tau }\right]}_{m}\;$ is the SINR for anchor *m*, and *K* is the number of antennas at anchor *m*. Additional parameters and further definitions are described in previous work (see Appendix A in [16] or [12]). With this, the contribution of delay measurements to the FIM for measurements in-between multiple anchors and all nodes of a cluster can be written as

(A7) $$J_{p_{c}}^{\left(\tau \right)}=\sum_{m=1}^{M}\sum_{l\in L}\Lambda \frac{8\pi^{2}}{c^{2}}\beta^{2}{\left[{\tilde{\mathrm{SINR}}}_{\tau }\right]}_{m}\;K\;R_{r}\left(\phi_{l,m},\vartheta_{l,m}\right),$$

where $R_{r}\left(\phi_{l,m},\vartheta_{l,m}\right)$ is the previously mentioned RDM, and Λ is the correction factor discussed in the following subsection. As argued in Section 5, an upper bound on this component is used in (25).

### Appendix B.2. Correction Factor Λ

As the delays $\tau_{m}\left(p_{l}\right)$ of each node in the cluster are in the vicinity of the delay $\tau_{m}\left(p_{c}\right)$, we can assume that $\tau_{m}\left(p_{l}\right)\approx \tau_{m}\left(p_{c}\right)\;\forall l\in L$. Still, small offsets remain. To address this, we introduce an equivalent log-likelihood of Equation (12) as

(A8) $$\ln f(r|\tau_{m}\left(p_{c}\right),\epsilon ,\eta ,\alpha )=\ln f(r_{c}|\tau_{m}\left(p_{c}\right),\alpha ),$$

with the assumption of parameters ϵ being estimated correctly and omitting parameters η of the DMC. The vector $r_{c}$ is a summed and weighted receive signal defined as

(A9) $$r_{c}=\sum_{l\in L}c_{l}^{*}r_{l},$$

with $c_{l}^{*}$ being complex conjugate weights that are introduced to maximize the SNR for $r_{c}$. The vector $r_{l}$ is the received signal from Equation (3), omitting indices *m* and *k* for the sake of easier notation. We now define a summed and weighted received signal without noise as

(A10) $$r_{c}^{\left(s\right)}=\sum_{l\in L}c_{l}^{*}s\left(\tau \left(p_{l}\right)\right)\alpha_{l,m}\approx s\left(\tau \left(p_{l}\right)\right)c^{H}\alpha ,$$

with $c={\left[c_{l}\right]}^{T}\;\forall l\in L$ being a stacked version of all weights within a cluster, where we assume the delays to be approximately equal for all l∈L. Furthermore, we introduce a weighted noise vector $r_{c}^{\left(n\right)}=\sum_{l\in L}c_{l}^{*}w_{w}$, again omitting indices m,k for easier notation. From this, we can define an SNR of this mean weighted signal as

(A11) $${\mathrm{SNR}}_{c}=\frac{∥r_{c}^{\left(s\right)}{∥}^{2}}{E\{∥r_{c}^{\left(n\right)}{∥}^{2}\}}\approx \frac{∥s\left(\tau_{l,m}\right){∥}^{2}\;{\left|c^{H}\alpha \right|}^{2}}{N_{s}\;\sigma_{n}^{2}\;{∥c∥}^{2}}.$$

Maximizing this SNR, we obtain

(A12) $${\mathrm{SNR}}_{c}^{\max}\approx \frac{{∥s(\tau \left(p_{c}\right)∥}^{2}\;{∥\alpha ∥}^{2}}{N_{s}\;\sigma_{l,m,k}^{2}},$$

which maximizes the inner product $c^{H}\alpha$ by setting the weights c=α. With this, we can see that the previous log-likelihood in (A8) is proportional to

(A13) $$\ln f(r_{c}|\tau_{l}\left(p_{c}\right),\alpha )\propto {∥r_{c}-\sum_{l\in L}{∥\alpha_{l}∥}^{2}s\left(\tau_{l,m}\right)∥}^{2}.$$

We can then express $\sum_{l\in L}{∥\alpha_{l}∥}^{2}s\left(\tau_{l,m}\right)=s_{c}$, which is a weighted summed baseband signal with delays $\tau_{m}\left(p_{c}\right)$. We introduce the Fourier transform of the delayed signal vector $s(\tau_{l})$ as $S\left(\tau_{l},f\right)=S\left(f\right)e^{-j2\pi f\tau_{l}}$. With this, we can write the squared sum of the time derivative of $s_{c}$ as

(A14) $$\begin{array}{cc} ∥{\dot{s}}_{c}{∥}^{2} & =\int_{f}{\left|\sum_{l\in L}{∥\alpha_{l}∥}^{2}S\left(f\right)e^{-j2\pi f\tau_{l}}\right|}^{2}f^{2}df\\ \; & =\int_{f}{\left|S\left(f\right)\right|}^{2}{\left|\sum_{l\in L}{∥\alpha_{l}∥}^{2}e^{-j2\pi f\tau_{l}}\right|}^{2}f^{2}df\\ & =\int_{f}{\left|S\left(f\right)\right|}^{2}{\left|\Lambda \left(f\right)\right|}^{2}f^{2}df \end{array}$$

where Λ(f) has a low-pass characteristic, reducing the effective bandwidth of S(f). We denote the effective reduction of the bandwidth by the factor Λ as

(A15) $${∥{\dot{s}}_{c}∥}^{2}=\Lambda {∥\dot{s}∥}^{2}=\Lambda \beta^{2}.$$

This correction factor Λ for the CRLB describing the relative bandwidth loss in a cluster with Λ∈[0,1], which corresponds to the factor Λ in Equation (25). This factor can also be interpreted as a mean information loss in a cluster due to mutual information within the clusters. Here, $\beta^{2}$ is the mean squared bandwidth of the signal, defined in Appendix C.

## Appendix C. Derivation the AEB

The angulation error bound (AEB) for a general angle ϕ representing either azimuth or elevation, the EFI for the case incorporating DM is approximated by [16]

(A16) $$J_{AEB}\left(\phi \right)\approx 8\pi^{2}\mathrm{SINR}\;M\;D_{\lambda }^{2}\left(\phi \right),$$

where $D_{\lambda }^{2}\left(\phi \right)$ is the normalized squared array aperture, which is defined as

(A17) $$D_{\lambda }^{2}\left(\phi \right)=\frac{1}{M}\sum_{m=1}^{M}\frac{d_{m}^{2}}{\lambda^{2}}\sin^{2}\left(\phi -\phi_{m}\right),$$

with $\phi_{m}$ being the angle of array elements relative to the coordinate system per anchor, and $d_{m}$ is the distance of array elements to the anchor position. With this, we can define the AEB for azimuth $\phi_{l,m}$ and elevation $\vartheta_{l,m}$ as $A_{\phi_{l,m}}=\sqrt{J_{\phi_{l,m}}^{-1}}$ and $A_{\vartheta_{l,m}}=\sqrt{J_{\vartheta_{l,m}}^{-1}}$. For the case of clustering, these bounds can then be found to be

(A18) $$\begin{array}{cc} A_{\phi_{m}} & =\sqrt{{\left[J_{\phi_{m}}\right]}^{-1}}=\sqrt{{\left[\sum_{l\in L}J_{\phi_{l,m}}\right]}^{-1}} \end{array}$$

(A19) $$\begin{array}{cc} A_{\vartheta_{m}} & =\sqrt{{\left[J_{\vartheta_{m}}\right]}^{-1}}=\sqrt{{\left[\sum_{l\in L}J_{\vartheta_{l,m}}\right]}^{-1}}, \end{array}$$

where $J_{\phi_{m}}$ and $J_{\vartheta_{m}}$ are the clustered EFIMs for azimuth and elevation, respectively. Note that here, no correction factor is required, as the equivalent aperture reduction is negligible in the case of the AEB due to $f_{c}^{2}\gg \left(\Lambda \beta^{2}\right)$ [16]. Furthermore, over a whole scenario, it can be assumed that $E_{l}\left[\phi_{l,m}\right]\approx 0$ and $E_{l}\left[\vartheta_{l,m}\right]\approx 0$, thus the dependency on different positions can be assumed to be negligible for the AEB. From this, we can define an EFI for a multi-anchor clustered case for azimuth and elevation as

(A20) $$J_{p_{c}}^{\left(\phi \right)}\approx \sum_{m=1}^{M}\sum_{l\in L}\frac{8\pi^{2}}{d_{l,m}^{2}}D_{\lambda }^{2}\left(\phi_{l,m}\right){\mathrm{SINR}}_{m}\;K\;R_{r}\left(\phi_{l,m}+\frac{\pi }{2},\vartheta_{l,m}\right),$$

(A21) $$J_{p_{c}}^{\left(\vartheta \right)}\approx \sum_{m=1}^{M}\sum_{l\in L}\frac{8\pi^{2}}{d_{l,m}^{2}}D_{\lambda }^{2}\left(\vartheta_{l,m}\right){\mathrm{SINR}}_{m}\;K\;R_{r}\left(\phi_{l,m},\vartheta_{l,m}+\frac{\pi }{2}\right),$$

with the previously mentioned RDM and an SINR per anchor as ${\mathrm{SINR}}_{m}$.
